# AGE-RELATED FUNCTIONAL RESERVE DECLINE IS NOT SEEN IN PHARYNGEAL SWALLOWING PRESSURES

**DOI:** 10.1093/geroni/igz038.613

**Published:** 2019-11-08

**Authors:** Corinne A Jones, Melanie Looper, Timothy McCulloch

**Affiliations:** 1 The University of Texas at Austin, Austin, Texas, United States; 2 University of Wisconsin - Madison, Madison, Wisconsin, United States

## Abstract

Age-related decline in functional reserve has been described in tongue strength: tongue pressure during swallowing does not change with age, but maximal-effort isometric tongue pressure decreases with age. Healthy persons show a slight increase in pharyngeal swallowing pressure with age, but it is unknown if there is a similar decline in functional reserve. Fifty-six healthy adults (n=38 60 years) underwent pharyngeal high-resolution manometry during effortful and normal-effort thin liquid swallows. Repeated measures ANOVAs were performed on maximum pressures, pharyngeal contractile integral (PCI), pharyngeal pressure gradients, and upper esophageal sphincter minimum pressures. We hypothesized that older individuals would generate a less-robust pressure increase with effortful swallowing than younger individuals. Maximum pressures, PCI, and gradients increase during effortful swallowing (p<0.001), but there was no interaction effect with age, suggesting a lack of age-related functional reserve decline. Older individuals had greater UES minimum pressures than younger individuals in the effortful swallowing task (p=0.03), which may stem from reduced muscular compliance in this area. These findings do not align with those reported in tongue pressures, suggesting that muscle properties and pressure generation may be fundamentally different between the pharynx and the oral tongue. Alternatively, the effortful swallowing task may not elicit maximum contractility of the pharyngeal musculature. The preserved ability to increase pharyngeal pressure during effortful swallowing may support the use of the effortful swallow exercise in older adults with swallowing disorders.

